# Investigating avian competition for surface water in an arid zone bioregion

**DOI:** 10.1002/ece3.10396

**Published:** 2023-08-03

**Authors:** Simon E. Votto, Christine Schlesinger, Fiona Dyer, Valerie Caron, Jenny Davis

**Affiliations:** ^1^ Research Institute for the Environment and Livelihoods Charles Darwin University Casuarina Northern Territory Australia; ^2^ Centre for Applied Water Science University of Canberra Bruce Australia Capital Territory Australia; ^3^ Health and Biosecurity Commonwealth Scientific and Industrial Research Organisation Black Mountain Australian Capital Territory Australia

**Keywords:** avian, interference competition, seasonal variation, temporal overlap, waterhole

## Abstract

Interference competition has the potential to alter avian assemblages at long‐lasting arid zone waterholes, particularly in a warming world, as more potentially aggressive species frequent these sites to drink. We used camera traps and observational surveys to investigate interference competition between terrestrial avian species at six long‐lasting waterholes across three sampling seasons (two summers and one winter) within the MacDonnell Ranges Bioregion in central Australia. The proportion of individuals drinking for each of four dietary classes (granivores, nectarivores, omnivores, and insectivores) was modelled in relation to their abundance in the immediate waterhole habitat, which informed the potential for competition in each season. We then used the temporal overlap estimators to quantify the degree of competition between species at waterholes with species grouped into families (Meliphagidae, Ptilonorhynchidae, Estrildidae, and Rhipiduridae). We found the proportion of individuals drinking at waterholes was greatest during hot and dry periods, suggesting the potential for interference competition is greatest during these times. This was particularly the case for nectarivores where, in hot and dry conditions, the proportion of drinking individuals increased significantly as their abundance also increased in the waterhole habitat. We predicted that subordinate species would alter their activity periods to avoid competitive interactions with meliphagids (honeyeaters), however, we found there was a high degree of temporal overlap between all families sampled across all seasons. These results suggest subordinate species are unlikely to be excluded from long‐lasting waterholes by potentially aggressive species, such as honeyeaters. However, some species may face trade‐offs between foraging and accessing waterholes to stay hydrated as they shift their activity to avoid the hottest parts of the day during the summer months. Under global warming, extended hot and dry periods will likely create conditions where balancing energy and hydration requirements becomes increasingly difficult and results in the loss of body condition.

## Graphical abstract

Interference competition has the potential to alter avian assemblages at long‐lasting arid zone waterholes. However, we found there was a high degree of temporal overlap between the species drinking at our study sites. Our results suggest subordinates are unlikely to be excluded from long‐lasting waterholes by aggressive honeyeaters.

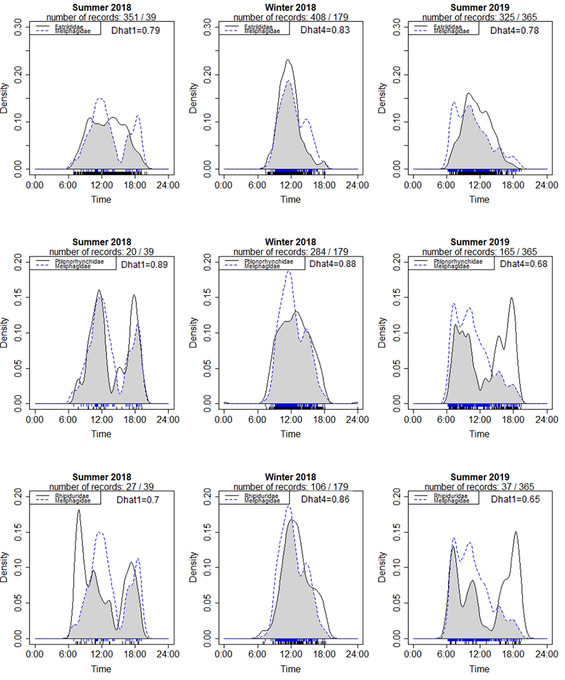

## 1 INTRODUCTION

Competition is an important ecological process that has the potential to alter avian assemblages (Giller, 1984; Maurer, 1984; Wiens, 1989; Ziv et al., 1993). Competitive processes between species are generally described as either exploitation competition (also known as scramble or depletion competition, where resources are removed by one species, leaving less for another) or interference competition (where the activities of one species prevent another from accessing a mutually desired resource; Maurer, 1984; Schenk, 2006). The frequency and intensity of competitive interactions between species have the potential to change over time, particularly in environments where seasonal conditions strongly influence the availability of and demand for mutually desired resources (Robinson, 1981; Srinivasan et al., 2018; Williams & Batzli, 1979). Climate change has also been shown to affect competition by altering the timing of seasonal changes or the population densities of competing species (Markus et al., 2007). Species that adapt quickly to these climatic changes generally have a competitive advantage over those that are slower to respond (Samplonius & Both, 2019).

Long‐lasting waterholes in arid zone environments are areas where interspecific and competitive interactions can occur, particularly during hot and dry periods, when surface water in the surrounding landscape is scarce. Both population densities and the time individuals spend in these habitats can increase during periods of water scarcity (Thrash et al., 1995), therefore increasing the likelihood of interference competition between species. Investigation of interference competition at waterholes has mainly focussed on large mammals in Africa (Sirot et al., 2016; Valeix et al., 2007, 2008, 2009). There has been less research on competitive processes between avian species at waterholes, however, see O'Reilly et al. (2019) and Attum et al. (2021).

Direct interference competition can be avoided by reducing the period of temporal overlap between competing species (Kronfeld‐Schor & Dayan, 2003). In this process, the subordinate species generally shift their activity around the dominant species, avoiding the times when and areas where they are present. Although avoidance behaviour allows subordinate species to reduce the frequency of competitive interaction, it may also result in longer‐term consequences if they are highly dependent on the resources from which they are being excluded (Bednekoff & Houston, 1994; Kennedy & White, 1996).

The dependence of avian species on surface water is generally related to the amount of water that can be derived from their diet (e.g. granivore, nectarivore, omnivore and insectivore; MacMillen & Baudinette, 1993; Merrick, 2006) and the environmental conditions of the habitats in which they reside. The relatively high moisture content of insectivore, nectarivore, and omnivore diets means that they are generally less dependent on waterholes to meet their water requirements (Dawson & Bartholomew, 1968; Fleming et al., 2004; Smit et al., 2019; Smyth & Coulombe, 1971) than granivores, which have a dry seed diet (Cade & Dybas, 1962; Zann & Bamford, 1996). However, in arid environments, air temperature is a significant environmental driver that affects the water consumption of avian species (McKechnie et al., 2017; McWhorter et al., 2018; Smit et al., 2018; Tieleman et al., 2003). Recent studies have found that, as air temperatures increase and the landscape dries out, even species that are relatively independent of surface water increase their rate of drinking at long‐lasting waterholes (Lee et al., 2017; Votto et al., 2020).

Some nectarivores, particularly honeyeaters, are known for their aggressive behaviour toward other species (Mac Nally & Timewell, 2005). Their aggression is often manifested in chasing behaviour and physical attacks, which are usually targeted toward species attempting to access high‐value and mutually desired resources, such as nectar and lerps (Davis & Recher, 1993; Woinarski, 1984). However, some honeyeaters will attempt to exclude most other species from their territory, regardless of the resources they may be accessing within it (Clarke, 1984). There is the potential that honeyeaters that establish a territory in a waterhole habitat may attempt to defend the water resources associated with them, excluding others from accessing them in the process. If the time (temporal niche breadth) honeyeaters spend at waterholes increases significantly during hot and dry periods, complete avoidance by other species heavily dependent on water (e.g., granivores) may not be possible.

Avian predators, such as raptors, also have the potential to exclude prey species from waterhole sites while they are accessing them. Votto et al. (2020) found that raptors significantly increased their frequency of drinking at waterholes as weather conditions became increasingly hot and dry. While raptors were present, other species were deterred from accessing the sites for periods of up to 50 min. As the presence of raptors at waterholes has previously been shown to exclude other birds from waterholes, we chose to focus on the competitive interactions between non‐predatory species in this study.

In this study, we investigate temporal overlaps between terrestrial avian species at long‐lasting waterholes in the arid central Australian ranges under varying seasonal conditions using camera traps and direct surveys. While camera traps are useful tools for continuously surveying focal points of activity, such as drinking at waterholes, they are unable to capture activity in the surrounding habitat. The greater soil moisture associated with waterhole habitats enables foliated plants, such as eucalypts and ferns, to flourish (Free et al., 2013; Porporato et al., 2002). These plants are important for a variety of birds in arid ecosystems as they provide shelter and shade (Funghi et al., 2019) and are associated with a variety of food resources, such as nectar and insects (Ford & Paton, 1985; Sheldon et al., 2010). Therefore, not all birds accessing waterhole sites are necessarily there to drink, some may instead be there to seek shade or to forage.

Direct surveys provided an opportunity to identify periods of high activity at the waterhole sites – when the potential for competition is highest – and the species most likely to be competing while approaching the water. The proportion of individuals within a dietary class observed drinking relative to their abundance in the immediate waterhole habitat provides an indication of their reliance on surface water. Groups that are reliant on surface water are likely to drink when present at waterholes (Smit et al., 2019), and therefore, more likely to compete for the resource when approaching.

We predicted that the proportion of individuals drinking at sampled waterholes would be greatest for: (a) granivores under all seasonal weather conditions; and (b) nectarivores and omnivores as weather conditions became increasingly hotter and drier. We did not anticipate changes in insectivore drinking under any seasonal weather conditions because of the higher water content of their diet. Finally, we predicted that selected species of granivores, omnivores, and insectivores – the Estrildidae (finches), Ptilonorhynchidae (bowerbirds), and Rhipiduridae (wagtails) – would shift their temporal niches to avoid competitive interactions with aggressive nectarivores – the Meliphagidae (honeyeaters) – as the number of meliphagids drinking at waterholes increased during hot and/or dry periods.

## 2 METHODS

### 2.1 Study area and sites

The MacDonnell Ranges Bioregion contains the largest collective group of mountains in central Australia and includes several distinct range systems. The ranges are rugged and rise to elevations of 1531 m above sea level (Mount Zeil). The climate in this bioregion is considered arid, but the mountains are high enough to generate greater average annual rainfall (although still highly variable) than experienced in the surrounding drylands and deserts (372 mm, Coefficient of Variation 61% vs. 188 mm, Coefficient of Variation 72%; Bastin, 2012). Many long‐lasting waterholes in this region are situated within sandy rivers and creek lines, such as the Finke River, and are often within, or adjacent to, rocky gorges (Duguid et al., 2005). Groundwater inputs support their persistence under drought conditions (Hatton et al., 1998).

**FIGURE 1:**
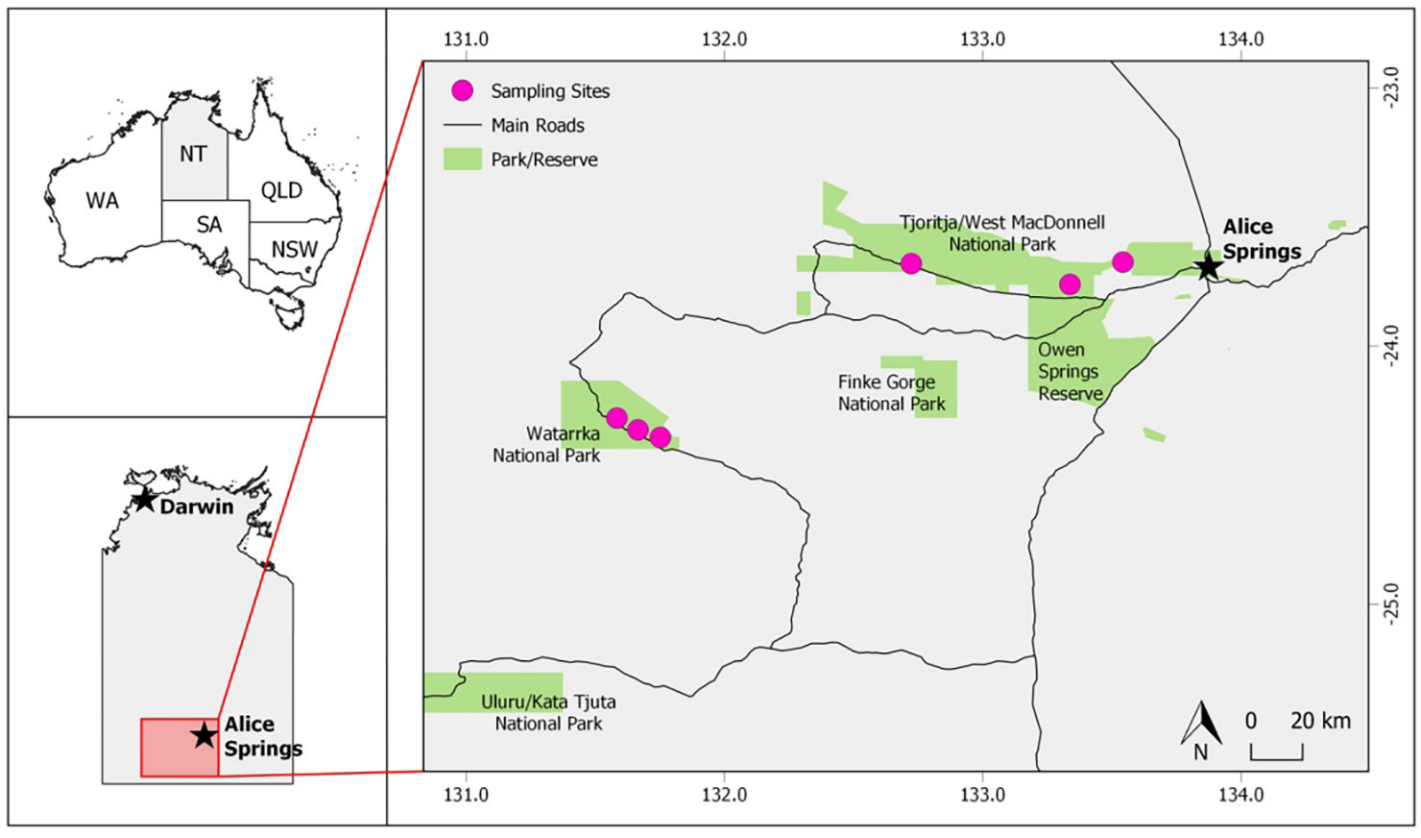
Locations of sampling sites and national parks within the Central Australian Ranges/MacDonnell Ranges Bioregion, Australia.

The study was conducted at six small (<600 m^2^) but long‐lasting waterholes. Here, we define long‐lasting as persisting for several years, even if minimal rainfall is received. Three sites were in the West MacDonnell Ranges, within the Tjoritja/West MacDonnell Ranges National Park and three were in the George Gill Range, within Watarrka National Park (Figure 1). All sites are located within the MacDonnell Ranges Bioregion, the former being within the MacDonnell Subregion and the latter within the Watarrka Subregion (Environment Australia, 2000). The sites were all characteristic of upland waterholes, as described by Duguid et al. (2005), however, their size and the structure and composition of the surrounding vegetation varied (Table 1).

**TABLE 1:** Location of study sites in the Central Australian Ranges/MacDonnell Ranges Bioregion, their key plant species, and the average canopy height and canopy cover associated with each site (sampled within 40 m of the water's edge).

Site name	Area of surface water (seasonal average; m^2^)	Subregion	Key plant species	Average canopy height (m)	Average canopy cover (%)
Jay Creek Fish Hole	376	West MacDonnell	River Red Gum (*Eucalyptus camaldulensis*) Buffel Grass (*Cenchrus ciliaris*) Silky Browntop (*Eulalia aurea*)	7	54
Reedy Rockhole	286	West MacDonnell	River Red Gum (*Eucalyptus camaldulensis*) Witchetty Bush (*Acacia kempeana*) Kangaroo Grass (*Themeda triandra*)	5	47
Pioneer Creek Waterhole	302	West MacDonnell	River Red Gum (*Eucalyptus camaldulensis*) Black Tea‐tree (*Melaleuca bracteata*) Witchetty Bush (*Acacia kempeana*) Buffel Grass (*Cenchrus ciliaris*)	7	20
Penny Springs	40	Watarrka	Ghost Gum (*Corymbia aparrerinja*) MacDonnell Ranges Cycad (*Macrozamia macdonnellii*) Witchetty Bush (*Acacia kempeana*) Swamp Shield‐fern (*Cyclosaurus interruptus*)	10	96
Wanya Rockhole	112	Watarrka	River Red Gum (*Eucalyptus camaldulensis*) Rock Fig (*Ficus brachypoda*) Witchetty Bush (*Acacia kempeana*) Kangaroo Grass (*Themeda triandra*)	11	65
Stokes Creek Waterhole	344	Watarrka	River Red Gum (*Eucalyptus camaldulensis*) Witchetty Bush (*Acacia kempeana*) Kangaroo Grass (*Themeda triandra*)	19	50

### 2.2 Direct surveys

Study sites were surveyed across three sampling seasons, summer 2018 (from January to the end of February), winter 2018 (from July to the end of August), and summer 2019 (from January to the end of February). Four direct (observational) surveys were conducted at each site within each sampling season; two surveys at the beginning of each sampling season and two at the end of the season, coinciding with the days that the cameras were either deployed or removed from the study sites. The two surveys (one morning survey between 7 am and 9 am and an afternoon survey between 3 pm and 5 pm) were conducted at times of the day when birds are known to be active at waterholes (Evans et al., 1985; Fisher et al., 1972; Lee et al., 2017).

At each site, surveys were conducted from a single vantage point that provided good visual coverage of the entire site and was set back far enough so as not to disturb birds approaching the water. All birds present at the site, up to 40 m from the water's edge (including those flying overhead), were identified to species, along with the time at which they were sighted, the number of individuals, and their behaviours (e.g., drinking, foraging, calling) were recorded. Species were identified from their appearance with reference to Pizzey et al. (2013) or via their call, which was verified using the eGuide by Morcombe (2011). In many cases, the sex and age of the study species were not easily distinguishable so these data were not collected as part of this study.

A 5‐min event window was applied to species observed via direct surveys. Once a particular species was recorded, a five‐minute window had to lapse before a record of the same species could be counted in the next independent sampling event (ISE). This enabled us to estimate the frequency of visits for a given species to the sampled waterhole sites and corresponded to analyses of camera data. Birds were classified into five dietary classes: insectivores, granivores, carnivores, insectivores or omnivores (Tischler et al., 2013), which were applied to the analysis investigating the proportion of individuals drinking at waterholes relative to their abundance in the surrounding habitat. Waterbirds (birds that are reliant on water as their primary habitat) were recorded at each site but removed from the subsequent analyses of both the observational and camera trap datasets, primarily because their use of and behaviour at long‐lasting waterholes differs from species that do not require water as habitat.

### 2.3 Camera trapping

Passive infrared (PIR) triggered (Reconyx HyperFire HC600 and Ltl Acorn 5210A) camera traps were used to record the presence of avifauna at each site. Camera traps were set up in a systematic manner (between 10 and 20 m apart depending on the size of the waterhole) to cover as much of the perimeter of the waterhole as possible. A total of 39 cameras were deployed across the sampling sites during each sampling period. The number of cameras varied per site from four to nine, depending on the size of the waterhole. Of the 39 cameras deployed across the sampling sites, six were Reconyx and 33 were Ltl Acorn. Reconyx cameras were used at sites where there were not enough Ltl Acorn cameras to provide full coverage, and no more than two Reconyx cameras were used at any one site.

Cameras were mounted on stakes and positioned approximately 1.5 m away from the water's edge and 30–40 cm above the ground. This distance and height were selected so that the cameras' PIR sensors were aligned parallel to the waterline, such that they were at the optimal position to detect both the small and large birds that accessed the study sites (Meek et al., 2012; Swann et al., 2004). Camera traps were set for a continuous period of 5 weeks across all sites and within each season. The passive infrared sensors on both camera models were set to high sensitivity with three images recorded per trigger event and no quiet period between triggers.

### 2.4 Image processing

Camera trap images were processed to the level of species using the open‐source application Camelot (v 1.5.4) and the method described by Hendry and Mann (2017). A trap event window of 5 min was applied to the dataset (Manzo et al., 2012; Villette et al., 2016, 2017; Votto et al., 2020), as was applied to the direct surveys. This means multiple images of the same species taken within the 5 min window were counted as a single independent trapping event (ITE). This was the case even if images of the same species were captured by multiple cameras within the same site simultaneously, although this happened rarely. When it did, the record for that time period was associated with the first camera site at which the species was detected.

We were unable to test for differences in activity periods between seasons by combining species at the dietary class level. This was because the activity periods for species within a given dietary class were often different (e.g., diurnal vs. crepuscular). Species within the same taxonomic family showed similar activity periods at waterholes. Therefore, select species from each dietary class were further sorted into their respective taxonomic families (Meliphagidae, Estrildidae, Ptilonorhynchidae, and Rhipiduridae) where camera trap data were used to analyse temporal overlap (Table S1).

### 2.5 Weather data

Mean maximum temperature and rainfall data for the Watarrka and Tjoritja/West MacDonnell Subregions (TWMSR) were obtained from the Australian Bureau of Meteorology 015652 (Watarrka; mean distance from the Watarrka sites = 13 km), 015667 (Ormiston Gorge; mean distance from the TWMSR sites = 51 km) and 015590 (Alice Springs Airport; mean distance from the TWMSR sites = 71 km) weather stations (Bureau of Meteorology, 2018). For TWMSR sites, we used rainfall data from Ormiston Gorge weather station, which was in closer proximity to the sites than the others, and temperature data from Alice Springs Airport weather station (as temperature data from the Ormiston weather station were not available). While rainfall recorded at the weather stations did not completely correspond with the rain that fell at the study sites – because rainfall in these environments is highly patchy – the data provide an accurate representation of weather conditions experienced at a regional scale, and relative dryness between seasons and years.

Air temperatures and rainfall varied substantially between sampling periods, as expected of an inland, mid‐latitude, arid region (Table S2). The summer 2018 sampling season was hot and punctuated by periods of substantial rainfall. The winter 2018 sampling season was relatively cool and dry. The summer 2019 sampling season was intensely hot and dry.

### 2.6 Data analysis

#### 2.6.1 Species accumulation curves

We used PRIMER v7 (Anderson et al., 2008) to plot the accumulating number of species observed against sampling effort (survey hours) via direct survey at a given site within a given sampling season. Samples collected from a given site and season were then randomly sampled 9999 times. The resulting curves for each of the 9999 samples were then averaged to give the smoothed species accumulation curves for each site and season.

#### 2.6.2 Generalised linear mixed models

For each dietary class, generalised linear mixed models (GLMMs) with binomial distributions were used to model the proportion of individuals drinking at waterholes in relation to those observed in the immediate waterhole habitat. Modelling was carried out following the method of Zuur et al. (2009) and Brooks et al. (2017b). The fixed covariates in each model included total ISEs per dietary class and season. Site was included as the random effect for each model. The glmmTMB package (v 1.1.2) within R (v 4.1.1) was used to fit the model to data from each dietary class (Brooks et al., 2017a; R Core Team, 2018). The model is of the form represented in Equation 1:

(1) $$\begin{array}{c} y_{\mathrm{ijn}}\sim \text{binomial}\left(n_{\mathrm{ijn}},p_{\mathrm{ijn}}\right)\\ \text{logit}\left(p_{\mathrm{ijn}}\right)=\beta_{s}+\sum_{h=1}^{p}\beta_{h}x_{\text{hijn}}+a_{n}\\ a_{n}\sim \text{Gaussian}\left(0,\sigma_{a}^{2}\right) \end{array}$$

where $y_{\mathrm{ijn}}$ is the number of ‘individuals drinking’ in $n_{\mathrm{ijn}}$ events from the *i*th sample (*i* = 1,…,35), and the *j*th dietary class (*j* = 1,…,4) within the *n*th site (*n* = 1,…,6). $y_{\mathrm{ijn}}$ follows a binomial distribution with mean *np*, and a variance of $\mathrm{np}\left(1-p\right)$. The logit of $p_{\mathrm{ijn}}$ is modelled by a regression equation with $\beta_{s}$ as the intercept for the *s*th season (*s* = 1,…,3), $\beta_{h}$ is the slope (regression coefficient) of the *h*th predictor (*h* = 1), $x_{\text{hijn}}$ is the is *i*th response of the *j*th avian dietary class within the *n*th site for the *h*th predictor. *a*_*n*_ is the site‐specific random effect, which is assumed to be normally distributed with mean 0 and variance *σ* ^2^.

Residual patterns and deviations from the expected binomial distributions were examined for each GLMM applied to our dataset using R package DHARMa (v 0.4.4; Hartig, 2017). Non‐linear patterns in the residuals were examined by plotting standardised residuals against model predictions (Figures [Link], [Link]), to determine if model accuracy varied in relation to increases or decreases in the fixed covariates. Deviations from the expected binomial distributions (KS test) along with tests for heteroscedasticity (Dispersion test) and outliers (Outlier test) were examined for each model using QQ plots.

The variance explained (or *R* ^2^) by GLMMs applied to avian dietary classes were determined following the methods of Nakagawa and Schielzeth (2013). This was achieved by computing two separate measures of *R* ^2^ within a given GLMM, the marginal *R* ^2^ and conditional *R* ^2^, applied to a dietary class. The marginal *R* ^2^ measures the variance explained by the fixed effects for a given dietary class. The conditional *R* ^2^ measures the variance explained by both fixed and random effects to give an overall model fit for each dietary class.

#### 2.6.3 Activity overlap

Camera trap data were used to estimate temporal overlap of the Meliphagidae in relation to members of Estrildidae, Ptilonorhynchidae, and Rhipiduridae. Temporal overlap was quantified using the R package, CamtrapR (v 2.2.0; Niedballa et al., 2016), following the methods of Ridout and Linkie (2009). Activity patterns from each family (sampled across all sites within a given season) were computed using kernel density estimation. A kernel density bandwidth parameter of 0.8 was applied where the smaller of the sample sizes (ITEs) between the two families was under 50, and a bandwidth parameter of 1 when both sample sizes were over 50. A measure of overlap between the estimated density estimations for each family class was calculated using two coefficient of overlap estimators provided in Ridout and Linkie (2009), labelled ${\hat{∆}}_{1}$ (Dhat1) and ${\hat{∆}}_{4}$ (Dhat4). We used Dhat1 for smaller sample sizes (under 50) and Dhat4 for larger sample sizes (over 50).

## 3 RESULTS

The two survey methods provided different but complementary datasets. A total of 62 species (excluding waterbirds) were recorded from 144 hours of direct observational surveys, which resulted in 7544 ISEs being recorded across all study sites and seasons. Camera traps recorded 49 species (excluding waterbirds), which were captured by 39 camera traps deployed for a total of 98,280 camera trap hours. Camera traps recorded more than 185,000 images and 14,119 ITEs from the six study sites across three sampling seasons (Table S1).

**FIGURE 2:**
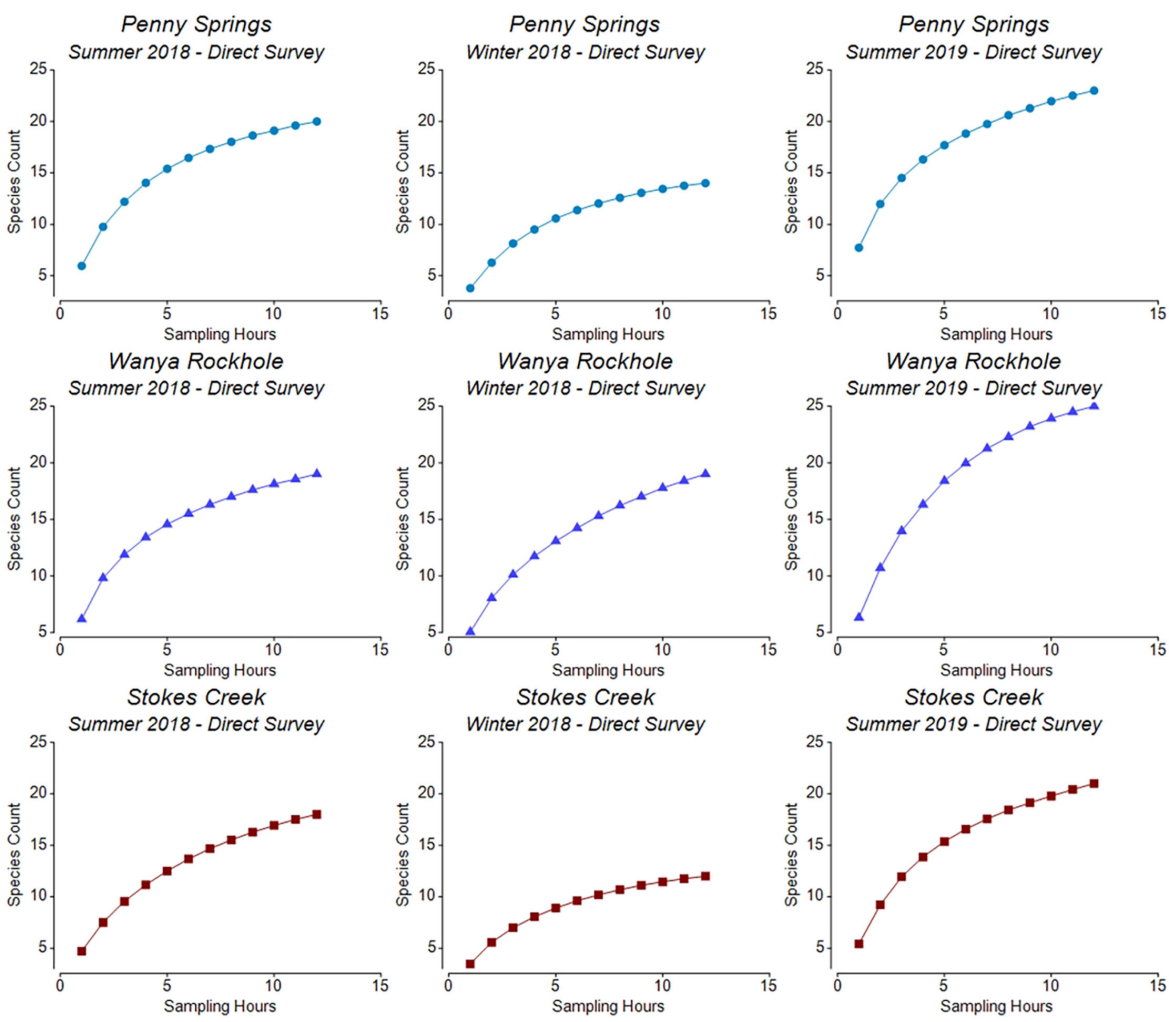
The smoothed rate of species accumulation in relation to sampling hours (sampled via direct survey) within each sampling season for the Watarrka National Park sampling sites.

**FIGURE 3:**
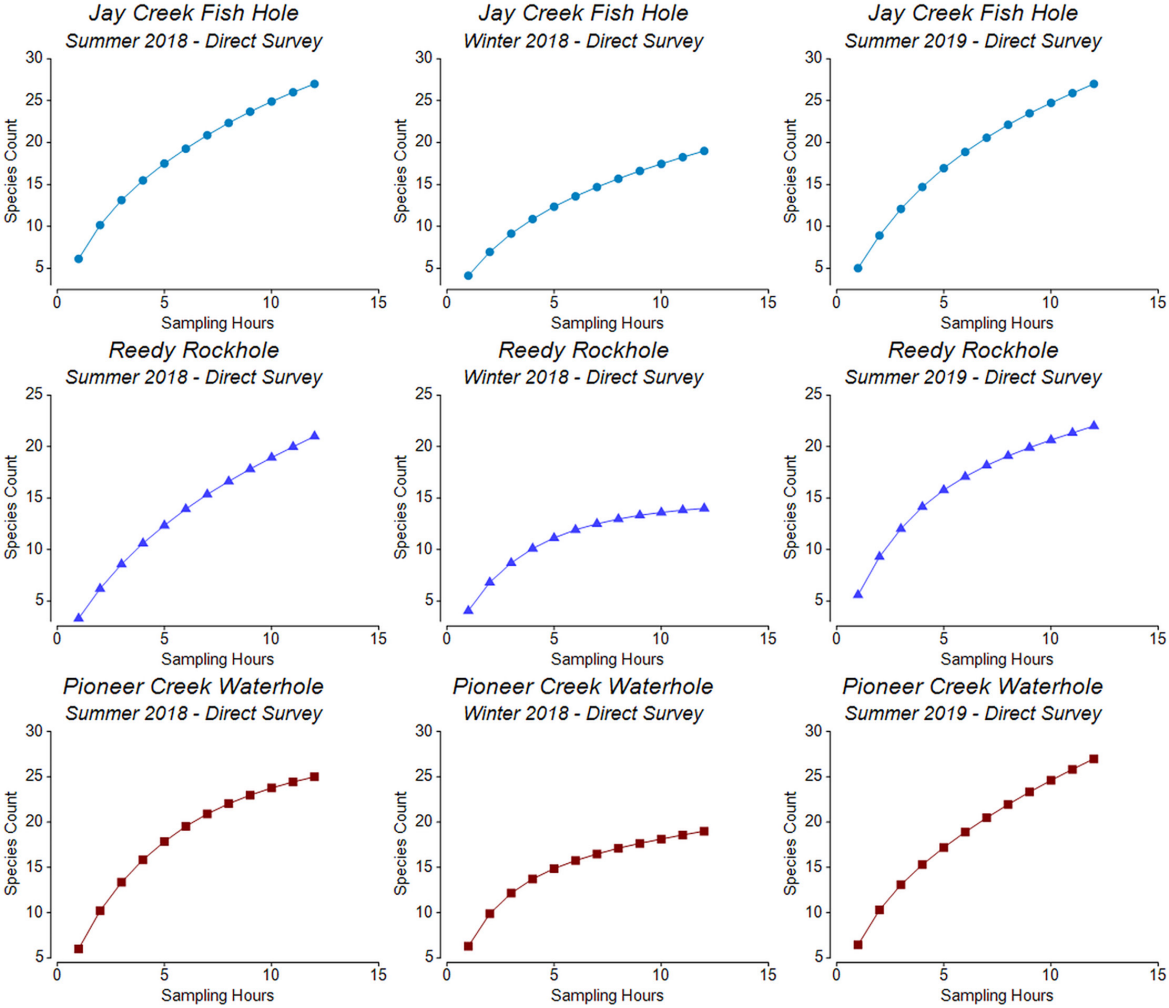
The smoothed rate of species accumulation in relation to sampling hours (sampled via direct survey) within each sampling season for the Tjoritja/West MacDonnell National Park sampling sites.

The seasonal rates of species accumulation were similar at waterholes across the MacDonnell Ranges Bioregion, with higher rates occurring during the summer seasons. Of the two summer seasons, accumulation rates were slightly greater during the hotter and drier summer 2019 season, and approximately five additional species were recorded across most sites at this time. Rates of accumulation were generally higher at the Tjoritja/West MacDonnell National Park sites, particularly during the summer 2019 season. Most of the smoothed curves did not reach an asymptote, particularly within the summer 2019 season, where accumulation rates were still increasing at the end of the sampling periods (Figures 2 and 3).

The proportion of individuals drinking at sampled waterholes within each dietary class differed significantly between seasons (model intercepts). The proportion of individuals drinking at sampled waterholes was significantly greater for all dietary classes in the summer 2019 season compared to the summer 2018 season. The proportion of granivores and omnivores drinking was also significantly greater during the winter 2018 season compared with the summer 2018 season. Granivores were the only dietary class where the greatest proportion of individuals drinking was observed during the winter 2018 sampling season (Table S3).

**FIGURE 4:**
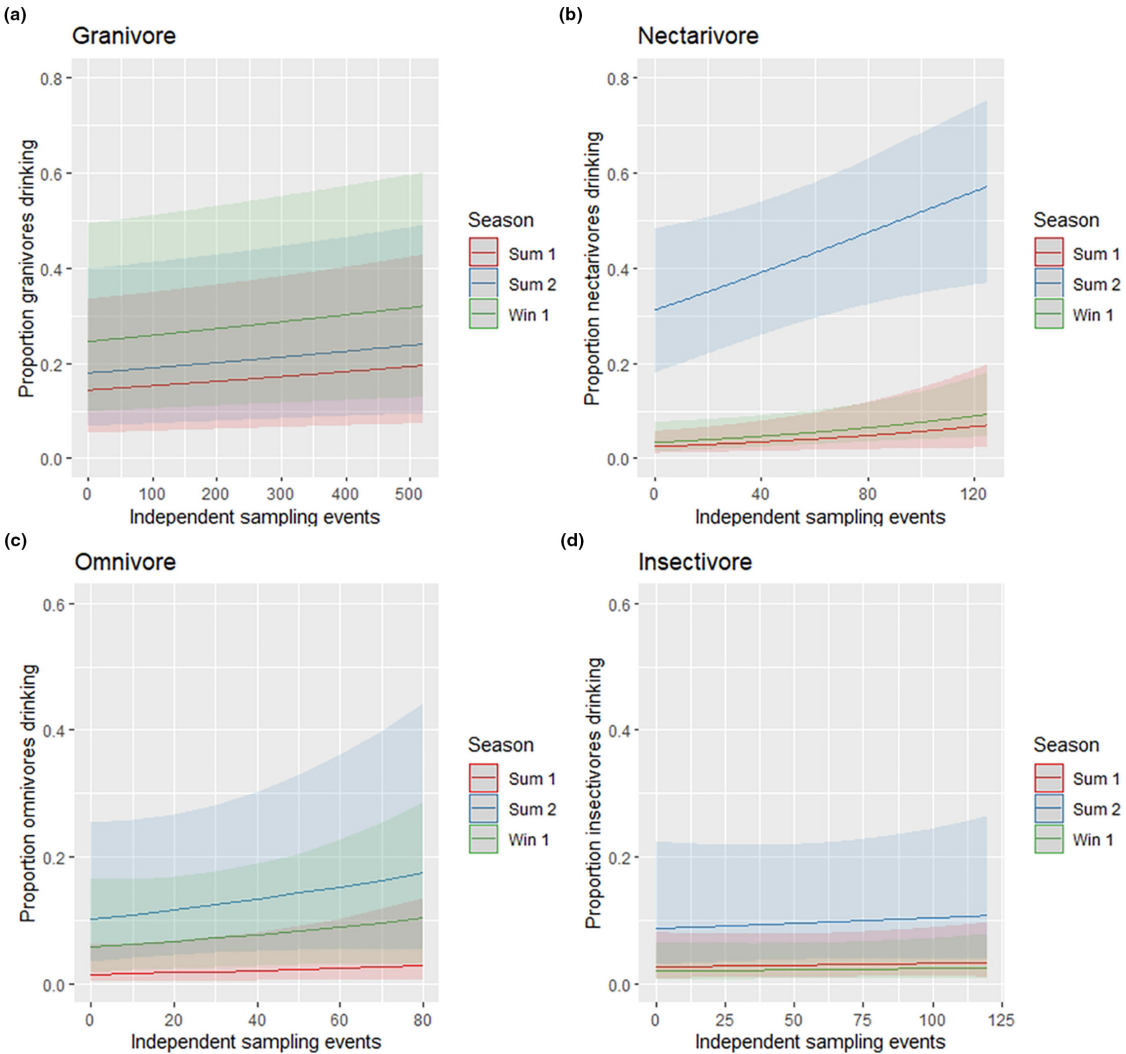
Model estimates with 95% confidence intervals for the proportion of individuals from each dietary class (granivores [a], nectarivores [b], omnivores [c] and insectivores [d]) drinking at sampled waterholes in relation to the mean independent sampling events for individuals observed in the immediate water habitat and season. Sum 1 = summer 2018; Sum 2 = summer 2019; Win 1 = winter 2018.

While the proportions of individuals drinking at waterholes varied significantly between seasons relative to those present in the surrounding habitat (not drinking), the slopes of the relationships between these variables (within seasons) were not significant in most cases. The exception was that during the summer 2019 season the proportion of nectarivores drinking increased significantly relative to the number of individuals (ISEs) sampled within the waterhole habitat. The relationship was linear and the proportion of individuals drinking increased from 30% to 60% as sampling events approached 120 (Figure 4).

**FIGURE 5:**
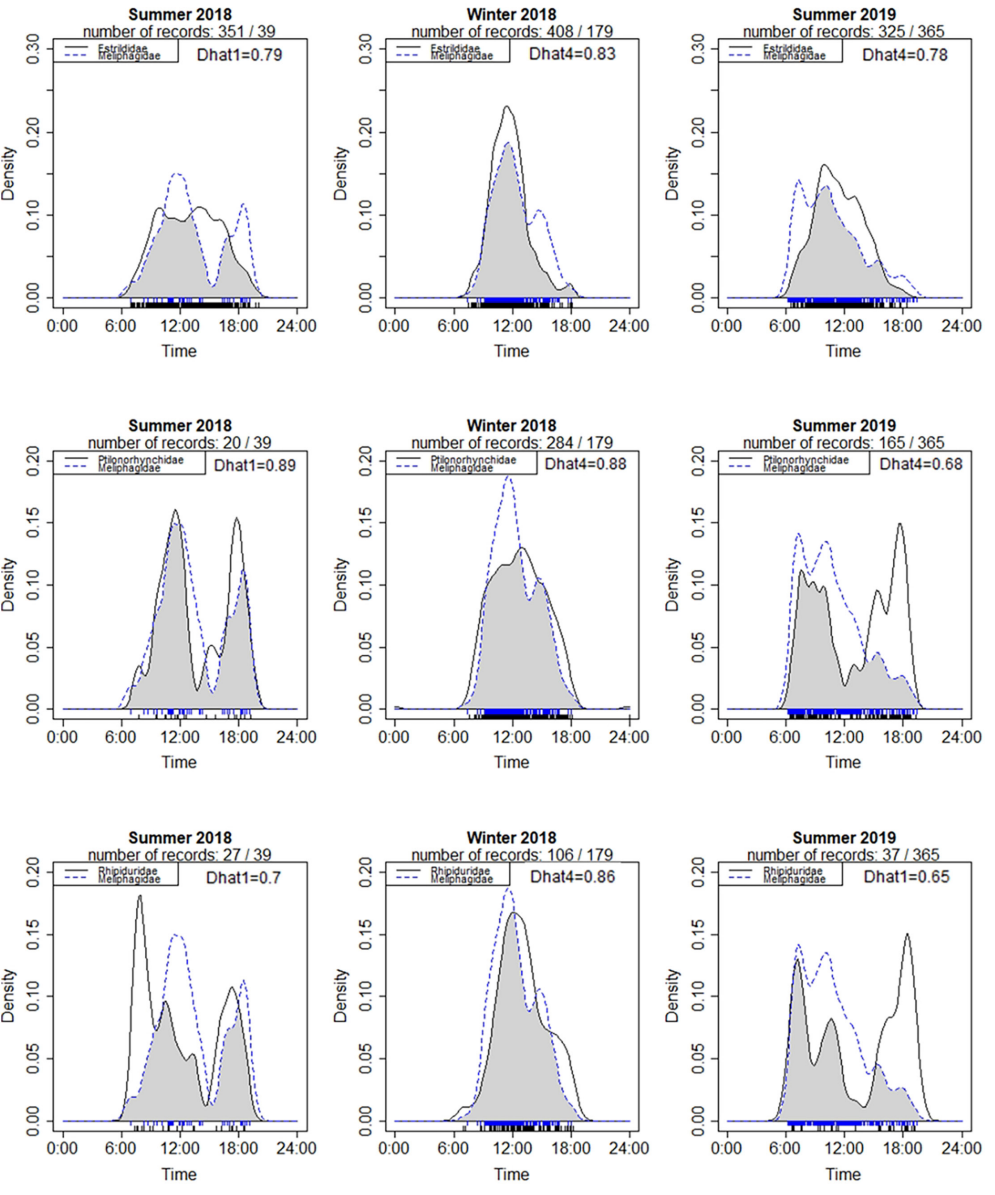
Estimates of the daily activity patterns of species within the Meliphagidae and their overlap with three potentially competitive family classes across each sampling site and season, based on camera data; Summer 2018, Winter 2018, and Summer 2019. The dashed lines are kernel‐density estimates for the Meliphagidae and the solid lines for the competing family classes. Each sample is indicated by the solid vertical line above the x‐axis. The coefficient of overlap is the area under the minimum of the two density estimates, as indicated by the shaded area in each plot and the Dhat values. The overlap estimator used is represented by Dhat1 or Dhat4.

The family classes, as sampled by camera traps, within this study displayed varied activity patterns across seasons. Estrildid (finch) species showed consistently similar patterns of activity across all seasons, with activity peaking at around 12 pm. Species within Ptilonorhynchidae and Rhipiduridae displayed similar activity patterns where, in the summer seasons, their activity densities were bimodal and peaked in the mornings and afternoons. The activity densities of Meliphagids were also bimodal during the summer 2018 season, however, they did not retain this pattern across the remaining seasons, adopting unimodal density pattens that peaked around 12 pm and declined steadily across the afternoon. Activity densities were similar (unimodal) for all sampled family classes, with their activity densities peaking around 12 pm (Figure 5).

The extent of overlap between estimated activity of the Meliphagidae and other families also varied across seasons. The greatest overlap between each family occurred during the winter season, where estimates ranged between 0.83 and 0.88. Activity overlap between Meliphagidae and Ptilonorhynchidae reduced in the summer seasons, with the summer 2019 season showing the greatest reduction in overlap as both families shifted to a bimodal activity distribution (overlap estimates 0.65 and 0.68). Meliphagidae overlap with the Estrildidae was consistently high across all sampling seasons, ranging from 0.78 to 0.83.

## 4 DISCUSSION

Our study examined surface water use and interference competition between avian species at arid zone waterholes under varying seasonal conditions. We found that the number of species present at waterholes was greatest in the second summer and of the individuals present, the proportion drinking was also greatest for most dietary classes at that time. These results were consistent with the increasing need for water under hot dry conditions. However, in many cases, as drinking activity increased, so did the degree of temporal overlap between family classes, indicating that interference competition may not be as important as originally predicted. This result was unexpected, particularly for nectarivores (Meliphagidae), which are known to display aggressive interactions with other species when foraging (Davis & Recher, 1993; Mac Nally & Timewell, 2005).

The greater rates of species accumulation in the summer seasons, compared to the winter season, was expected and likely reflects nomadic and migratory species seeking out longer‐lasting waterholes as the number of alternate water sources in the landscape is reduced and the metabolic requirements for water increase (Votto et al., 2022). However, we were surprised that rates of accumulation between the two summers were similar, given the greater amount of rain received in the first summer (~106 mm [summer 2018] vs. ~10 mm [summer 2019]). We expected that the number of species observed and the rate of accumulation would have been less in the wetter summer. This is because during the wet summer season, we assumed there would have been a greater number of temporary water sources and flowering plants in the broader landscape for birds to exploit, allowing them to be less reliant on long‐lasting sites. Our results suggest that long‐lasting waterhole sites are used by a similar number of species in wetter summers as they are in drier summers. This use may be more strongly influenced by access to food resources for many species, rather than access to water.

While the mean proportion of drinking individuals present within each dietary class generally increased across seasons as conditions became drier and hotter, the rate of increase in drinking individuals within a given season did not, in most cases. This result was surprising, particularly for granivores, given their strong surface water dependence. The reduction in drinking observed in the hot and dry summer may be related to changes in their behaviour and foraging activity under these conditions. Funghi et al. (2019) found that Zebra Finches drastically reduced their foraging activity, particularly at sites that were far from water, as air temperatures exceeded 35°C. The reduction in time spent foraging related to significant increases in heat dissipation behaviour (e.g., panting and wing‐lifting) observed under hot weather conditions. Shade, a resource that is generally associated with arid zone waterholes because of the abundance of foliated plants near the waterhole's edge (Free et al., 2013), is also sought more often by birds during hot conditions to minimise their heat load (Pattinson et al., 2020). While drinking is clearly important for granivores during hot and dry conditions, the apparent increases in their heat dissipation/minimisation behaviours may reduce their daily energy expenditure and reliance on surface water.

The exception in this study was nectarivores within the summer 2019 season. Our model had predicted that as the number of individuals increased within the immediate waterhole habitat, up to 60% would be drinking. These predictions were consistent with previous evidence that showed nectarivores increased their visitation to waterholes under similar conditions (Votto et al., 2020). Gardner et al. (2016) found that White‐plumed Honeyeaters (*Lichenostomus penicillatus*) suffered reductions in body condition in high temperatures, particularly under repeat exposure to daily maximum temperatures over 35°C. However, this decline was only found under low‐rainfall conditions when no rain had fallen for a period of 30 days or more.

The increased proportions of nectarivores drinking at waterholes during the summer 2019, when the proportion of individuals drinking within most other groups also increased, provided the opportunity to investigate whether interference competition increased under these conditions. However, we acknowledge some limitations with these findings primarily relating to the temporal and spatial coverage of the direct surveys.

Direct surveys were conducted at the time of day we considered birds would be most active at waterholes (Fisher et al., 1972), but based on camera trap results, in winter 2018, the drinking activity of many species peaked around 12 pm. The lack of direct survey data for the time of day when activity peaked in that season may have reduced the accuracy of our reported model outputs. In addition, we emphasise that our investigations of drinking behaviour in relation to relative abundance only relate to the abundance of birds at the waterhole, not in the wider landscape. A more accurate estimation of the surface water dependence of a given dietary class would require estimations of abundance of birds in the wider landscape, however, this was beyond the scope of our research.

We predicted that species such as granivores would shift the times they accessed waterhole sites to avoid nectarivores, considering the latter group are potentially aggressive. However, we found there were high degrees of overlap between the Meliphagidae, Estrildidae, and Ptilonorhynchidae, particularly during the summer and winter 2018 seasons. While the degree of temporal overlap was reduced between the Meliphagidae, Ptilonorhynchidae, and Rhipiduridae during the summer 2019 season, the high degree of overlap observed between these families during the summer and winter 2018 seasons suggests that these reductions were not likely related to interference competition.

The lack of interference competition between families at waterholes may relate to their perception that surface water is not a limited resource, unlike many food items, which can be depleted during the foraging process. Nectar is one such food item that can be depleted in individual flowers as birds access them. It is for this reason that nectarivores often forage for nectar early in the morning when the resource is at its highest (Bond & Brown, 1979). Depending on the number of flowering plants available for nectarivores, the nectar resource can be depleted by late morning/early afternoon (Ford, 1979). This increases the likelihood of interference competition occurring between nectivorous species, as competing individuals aggressively seek to obtain as much of the nectar resource as they can before it is depleted. The increased activity levels and aggression shown by nectarivores when foraging may increase their water requirements (particularly in hot conditions), which could explain why we observed them drinking during periods when they would also be foraging (i.e., during the morning).

Unlike nectar, surface water from waterholes is generally not depleted while individuals are drinking, so the perception is likely to be that it is an unlimited resource. Although it is in high demand during increasingly dry and hot conditions, the perception of an unlimited resource probably reduces the need for some individuals to exclude others from it. In fact, it may be beneficial for species to participate in mixed flocks while accessing water sites, as they are known to be hotspots for avian predators such as Collared Sparrowhawks (*Accipiter cirrocephalus*) and Brown Goshawks (*Accipiter fasciatus*; Fisher et al., 1972). Both of these avian predators frequent the waterholes sampled in this study, particularly under increasingly dry conditions where the daily maximum temperatures exceed 35°C (Votto et al., 2020). Accessing waterholes in mixed flocks could benefit individuals by increasing the probability of detecting an attacking predator and decreasing individual risk (Beauchamp & Livoreil, 1997; Siegfried & Underhill, 1975). These benefits may outweigh the increased risk of encountering a predator, when group size increases and attracts predator attention (Sorato et al., 2012). However, we note that mixed group foraging and predation risk is a complex topic that requires further study to gain a better understanding of these processes at waterholes.

The observed shifts in daily activity patterns displayed by the Ptilonorhynchidae, Rhipiduridae, and to a lesser extent Meliphagidae, were likely related to changes in weather conditions across seasons rather than interference competition, given the high degree of temporal overlap between the activity of different families. The shift to either bimodal activity (Ptilonorhynchidae and Rhipiduridae) or distributions skewed toward the early morning (Meliphagidae) in the hot summer months suggests the species in these families modify their behaviour to avoid activity during the hottest part of the day. Other studies have shown that avian foraging rates can decline as air temperatures become exceedingly hot (Edwards et al., 2015) and that many species in arid environments choose to shelter during these times to avoid overheating (du Plessis et al., 2012).

It appears that the cooler winter temperatures experienced during this study may have allowed some species to forage more efficiently in the morning without needing to access waterholes until later in the day. However, morning foraging activity in cooler conditions is dependent on the species, as some are less active during this time because it is either too cold or their key food items are not active (e.g., insects; Robbins, 1981). The shift toward early morning drinking activity shown by some species at waterholes during the warmer months may have implications for their fitness. The fast metabolic rate of birds means they generally lose body mass overnight and morning foraging is required to regain what is lost (Webster, 1989). Accessing waterholes earlier in the day potentially creates a trade‐off between gaining enough energy via foraging to regain body mass lost overnight and getting enough fluid to stay hydrated. Increased time spent drinking and engaging in other heat dissipation/minimisation behaviours at waterholes during periods usually spent foraging can result in losses in body condition for some species as they are unable to spend enough time foraging to regain overnight losses in body mass (du Plessis et al., 2012; Smit & McKechnie, 2015).

Interactions with raptors and even other non‐predatory animals may also affect when the target species of this study access waterholes For example, Brim Box et al. (2019) found the frequency of bird visits to arid zone waterholes was reduced on days camels (*Camelus dromedarius*) were present. Camels are particularly prevalent in arid Australia outside of managed national parks. As all the study sites sampled as a part of our study were within national parks, we did not detect any camels at the study sites. Raptors on the other hand were present at the waterholes at some times, and the frequency of their visits was highest during the hot and dry summer of 2019. The diel activity of ambush predators including the Brown Goshawk and Collared Sparrowhawk was highest during the middle of the afternoon (Figure S5). Their increased presence may have contributed to prey species such as the Western Bowerbird – a species within a weight range likely to be targeted by the Brown Goshawk (Olsen et al., 1990, 2018) – adjusting their activity at waterholes accordingly in the dry summer months.

The complexity of biological systems means there are always unmeasured variables and trends that are therefore unaccountable (Møller & Jennions, 2002). We consider that variables including sex (e.g., the proportion of males vs. females), age (e.g., adults vs. juveniles and sub‐adults) and the availability of food resources (e.g., nectar) associated with particular groups of birds could affect competitive interactions at waterholes, but we were not able to measure these variables. For example, feeding and breeding territories used by honeyeaters are aggressively defended by males, and their aggressiveness is at its highest when nectar is available, but only in moderate quantities (Armstrong, 1992). We predict the collection of demographic data would provide further insights into interspecific competition at waterholes between honeyeaters and other avian species.

## 5 CONCLUSIONS AND IMPLICATIONS FOR AVIAN CONSERVATION UNDER A WARMING CLIMATE

The generally high degree of overlap between species visiting long‐lasting waterholes in arid environments suggests that these sites are relatively communal places for non‐predatory birds. This may be due to a lack of perceived or actual scarcity in the surface water resource being accessed. Some species may also perceive a lower level of predation risk if they are accessing waterholes in mixed flocks. These results suggest potentially good outcomes for subordinate species in the future, where under global warming scenarios of high temperatures and prolonged drought, they are unlikely to be excluded from accessing water by other birds that often show aggressive behaviour while foraging for food. However, the effects of a warming climate on these bird assemblages may prevail. Under hot conditions, many species face trade‐offs between balancing foraging to meet their daily energy requirements and accessing waterholes to stay hydrated. Balancing their energy and hydration requirements may become more difficult under a warming climate if the number of hot days where early morning drinking is needed by these species increases. Longer durations in these sorts of conditions may result in these species losing body condition and fitness. We need to know more about the complex interactions between the behaviour, physiology, and dietary requirements of arid zone birds, in order to predict which species are most likely to be negatively affected by a warming climate.

## AUTHOR CONTRIBUTIONS

**Simon E. Votto:** Conceptualization (equal); formal analysis (lead); funding acquisition (equal); investigation (lead); methodology (lead); project administration (lead); writing – original draft (lead); writing – review and editing (lead). **Christine Schlesinger:** Conceptualization (supporting); formal analysis (supporting); funding acquisition (supporting); investigation (supporting); methodology (supporting); resources (supporting); supervision (supporting); writing – review and editing (supporting). **Fiona Dyer:** Conceptualization (equal); investigation (supporting); methodology (supporting); resources (supporting); supervision (supporting); writing – review and editing (supporting). **Valerie Caron:** Conceptualization (supporting); funding acquisition (supporting); methodology (supporting); supervision (supporting); writing – review and editing (supporting). **Jenny Davis:** Conceptualization (equal); formal analysis (supporting); funding acquisition (equal); investigation (supporting); methodology (supporting); resources (lead); supervision (lead); writing – review and editing (supporting).

## 6 FUNDING INFORMATION

This study was funded by an Australian Postgraduate Award and a generous donation from an anonymous donor (see acknowledgements). No external research grants were received for this study.

## Supporting information


Appendix S1

Figure S1.

Figure S2.

Figure S3.

Figure S4.

Figure S5.

## Data Availability

The data that support the findings of this study are openly available in Mendeley Data at http://doi.org/10.17632/jwkrhcknmw.1.
