# Supplementary material for: Investigating avian competition for surface water in an arid zone bioregion
Source: Ecol Evol. 2023 Aug 3;13(8):e10396. doi: 10.1002/ece3.10396 (PMC10400276; doi:10.1002/ece3.10396)
Supplement: Supplementary file 1 — Appendix S1 [file ECE3-13-e10396-s005.docx]

## Appendix

Appendix Table S1. Terrestrial avifauna recorded during the sampling period, their corresponding dietary class and the survey method used to identify them: direct survey (DS), camera trap (CT), both direct survey and camera trap (DS/CT). Species marked with * were included in the temporal overlap analysis.

| **FAMILY** | **SPECIES** | **COMMON NAME** | **DIETARY CLASS** | **SURVEY** |
| --- | --- | --- | --- | --- |
| **Acanthizidae** | *Acanthiza chrysorrhoa* | Yellow-rumped Thornbill | Insectivore | DS |
|  | *Smicrornis brevirostris* | Weebill | Insectivore | DS |
| **Acrocephalidae** | *Acrocephalus australis* | Australian Reed-warbler | Insectivore | DS |
| **Artamidae** | *Artamus cinereus* | Black-faced Woodswallow | Insectivore | DS |
|  | *Artamus minor* | Little Woodswallow | Insectivore | DS |
|  | *Artamus personatus* | Masked Woodswallow | Insectivore | DS/CT |
|  | *Cracticus tibicen* | Australian Magpie | Omnivore | DS/CT |
|  | *Cracticus nigrogularis* | Pied Butcherbird | Omnivore | DS/CT |
| **Cacatuidae** | *Calyptorhynchus banksii* | Red-tailed Black-cockatoo | Granivore | DS/CT |
|  | *Cacatua leadbeateri* | Major Mitchell’s Cockatoo | Granivore | DS/CT |
| **Campephagidae** | *Coracina novaehollandiae* | Black-faced Cuckoo-shrike | Insectivore | DS/CT |
|  | *Colluricincla harmonica* | Grey Shrike-thrush | Omnivore | DS/CT |
| **Columbidae** | *Phaps chalcoptera* | Common Bronzewing | Granivore | DS/CT |
|  | *Geophaps plumifera* | Spinifex Pigeon | Granivore | DS/CT |
|  | *Geopelia cuneata* | Diamond Dove | Granivore | DS/CT |
|  | *Ocyphaps lophotes* | Crested Pigeon | Granivore | DS/CT |
| **Corvidae** | *Corvus orru* | Torresian Crow | Omnivore | DS/CT |
| **Cuculidae** | *Chalcites basalis* | Horsfield's Bronze-cuckoo | Insectivore | DS |
|  | *Chalcites osculans* | Black-eared Cuckoo | Insectivore | DS |
| **Estrildidae** | *Taeniopygia castanotis* | Zebra Finch* | Granivore | DS/CT |
|  | *Emblema pictum* | Painted Finch* | Granivore | DS/CT |
| **Halcyonidae** | *Todiramphus sanctus* | Sacred Kingfisher | Omnivore | DS/CT |
| **Hirundinidae** | *Cheramoeca leucosterna* | White-backed Swallow | Insectivore | DS |
|  | *Petrochelidon ariel* | Fairy Martin | Insectivore | DS |
| **Locustellidae** | *Cincloramphus cruralis* | Brown Songlark | Insectivore | DS |
| **Maluridae** | *Malurus splendens* | Splendid Fairy-wren | Insectivore | DS/CT |
|  | *Malurus lamberti* | Variegated Fairy-wren | Insectivore | DS/CT |
| **Megaluridae** | *Poodytes gramineus* | Little Grassbird | Insectivore | CT |
|  | *Cincloramphus mathewsi* | Rufous Songlark | Insectivore | DS/CT |
| **Meliphagidae** | *Gavicalis virescens* | Singing Honeyeater* | Nectarivore | DS/CT |
|  | *Ptilotula keartlandi* | Grey-headed Honeyeater* | Nectarivore | DS/CT |
|  | *Ptilotula penicillatus* | White-plumed Honeyeater* | Nectarivore | DS/CT |
|  | *Ptilotula flavescens* | Yellow-tinted Honeyeater* | Nectarivore | DS/CT |
|  | *Sugamel niger* | Black Honeyeater* | Nectarivore | DS/CT |
|  | *Certhionyx variegatus* | Pied Honeyeater* | Nectarivore | DS/CT |
|  | *Purnella albifrons* | White-fronted Honeyeater* | Nectarivore | DS/CT |
|  | *Manorina flavigula* | Yellow-throated Miner* | Nectarivore | DS/CT |
|  | *Acanthagenys rufogularis* | Spiny-cheeked Honeyeater* | Nectarivore | DS/CT |
|  | *Lichmera indistincta* | Brown Honeyeater* | Nectarivore | DS/CT |
|  | *Melithreptus gularis* | Black-chinned Honeyeater* | Nectarivore | DS |
| **Meropidae** | *Merops ornatus* | Rainbow Bee-eater | Insectivore | DS/CT |
| **Monarchidae** | *Grallina cyanoleuca* | Magpie-lark | Insectivore | DS/CT |
| **Nectariniidae** | *Dicaeum hirundinaceum* | Mistletoebird | Nectarivore | DS/CT |
| **Otididae** | *Ardeotis australis* | Australian Bustard | Omnivore | DS |
| **Pachycephalidae** | *Pachycephala rufiventris* | Rufous Whistler | Insectivore | DS |
| **Pardalotidae** | *Pardalotus rubricatus* | Red-browed Pardalote | Insectivore | DS |
|  | *Pardalotus striatus* | Striated Pardalote | Insectivore | DS |
| **Pomatostomidae** | *Pomatostomus temporalis* | Grey-crowned Babbler | Insectivore | DS |
| **Psittacidae** | *Barnardius zonarius* | Australian Ringneck | Granivore | DS/CT |
|  | *Melopsittacus undulatus* | Budgerigar | Granivore | DS/CT |
|  | *Neopsephotus bourkii* | Bourke’s Parrot | Granivore | CT |
|  | *Psephotellus varius* | Mulga Parrot | Granivore | DS/CT |
| **Ptilonorhynchidae** | *Chlamydera guttata* | Western Bowerbird* | Omnivore | DS/CT |
| **Rhipiduridae** | *Rhipidura albiscapa* | Grey Fantail | Insectivore | DS |
|  | *Rhipidura leucophrys* | Willie Wagtail* | Insectivore | DS/CT |

Appendix Table S2. Variation in weather conditions including air temperature (maximum daily, mean maximum) and rainfall (maximum daily, total seasonal) recorded across each sampling season within each national park.

| **Season** | **National Park** | **Maximum daily temperature (°C)** | **Mean maximum temperature (°C)** | **Maximum daily rainfall (mm)** | **Total seasonal rainfall (mm)** |
| --- | --- | --- | --- | --- | --- |
| Summer 2018 | Tjoritja/West MacDonnell | 43 | 38.8 | 28 | 97.7 |
| Summer 2018 | Watarrka | 41 | 38.5 | 26.9 | 106.9 |
| Winter 2018 | Tjoritja/West MacDonnell | 31 | 23.5 | 2 | 3.3 |
| Winter 2018 | Watarrka | 30.1 | 24.9 | 9 | 12 |
| Summer 2019 | Tjoritja/West MacDonnell | 45.6 | 39.5 | 9 | 10 |
| Summer 2019 | Watarrka | 45.5 | 40.6 | 5 | 10.9 |

Appendix Table S3. Generalised linear mixed model output (log) for each dietary class (dependent variables). The sample size for each dietary class is represented by their independent sampling events (ISEs). The fixed effects section details model intercepts for each season and model slopes for the proportions of each dietary class drinking (*estimate*) along with their respective standard errors, and p-values. The random effects section details the variance for the random effect (site). The R^2^ section details the variance explained by the marginal (fixed effects only) and conditional (fixed and random effects) models.

|  | **Granivore (ISEs = 724)** | | | | **Omnivore (ISEs = 493)** | | | | **Nectarivore (ISEs = 846)** | | | | **Insectivore (ISEs = 595)** | | | |
| --- | --- | --- | --- | --- | --- | --- | --- | --- | --- | --- | --- | --- | --- | --- | --- | --- |
| *Fixed Effects* | *Estimate* | | *Std. Error* | *p* | *Estimate* | | *Std. Error* | *p* | *Estimate* | | *Std. Error* | *p* | *Estimate* | | *Std. Error* | *p* |
| (Intercept) [Sum 1] | -1.78 | | 0.56 | **0.001** | -4.16 | | 0.73 | **<0.001** | -3.67 | | 0.44 | **<0.001** | -3.59 | | 0.59 | **<0.001** |
| Season [Sum 2] | 0.26 | | 0.09 | **0.005** | 1.98 | | 0.55 | **<0.001** | 2.88 | | 0.36 | **<0.001** | 1.24 | | 0.28 | **<0.001** |
| Season [Win 1] | 0.66 | | 0.12 | **<0.001** | 1.37 | | 0.57 | **0.016** | 0.31 | | 0.42 | 0.462 | -0.30 | | 0.38 | 0.440 |
| Granivore.ISE | 0.00 | | 0.00 | 0.087 |  | |  |  |  | |  |  |  | |  |  |
| Omnivore.ISE |  | |  |  | 0.01 | | 0.01 | 0.342 |  | |  |  |  | |  |  |
| Nectarivore.ISE |  | |  |  |  | |  |  | 0.01 | | 0.00 | **0.031** |  | |  |  |
| Insectivore.ISE |  | |  |  |  | |  |  |  | |  |  | 0.00 | | 0.00 | 0.648 |
| *Random Effects* | | | | | | | | | | | | | | | | |
| σ^2^ | | 3.29 | | | | 3.29 | | | | 3.29 | | | | 3.29 | | |
| Site | | 1.80 _Site_ | | | | 1.16 _Site_ | | | | 0.46 _Site_ | | | | 1.17 _Site_ | | |
| *R^2^* | |  | | | |  | | | |  | | | |  | | |
| Marginal R^2^ / Conditional R^2^ | | 0.014 / 0.362 | | | | 0.150 / 0.371 | | | | 0.329 / 0.411 | | | | 0.091 / 0.330 | | |

### Model Validation

No clear non-linear patterns were found between the standardised residuals and model predictions for each model. There were no significant deviations from the binomial distributions (KS test p > 0.05), no heteroscedasticity (Dispersion test p > 0.05) and no outliers (Outlier test p > 0.05) detected within models associated with each dietary class (Appendix Figures 1–4).

Appendix Figure S1. QQ and residual plots for the granivore GLMM. A: QQ-plots of observed quantiles versus those expected by the negative binomial distribution. Within the QQ plot are tests for deviations from the negative binomial distribution (KS test), heteroscedasticity (Dispersion test) and outliers (Outlier test). B: Standardised residuals plotted against the model predictions.

Appendix Figure S2. QQ and residual plots for the omnivore GLMM. A: QQ-plots of observed quantiles versus those expected by the negative binomial distribution. Within the QQ plot are tests for deviations from the negative binomial distribution (KS test), heteroscedasticity (Dispersion test) and outliers (Outlier test). B: Standardised residuals plotted against the model predictions.

Appendix Figure S3. QQ and residual plots for the nectarivore GLMM. A: QQ-plots of observed quantiles versus those expected by the negative binomial distribution. Within the QQ plot are tests for deviations from the negative binomial distribution (KS test), heteroscedasticity (Dispersion test) and outliers (Outlier test). B: Standardised residuals plotted against the model predictions.

Appendix Figure S4. QQ and residual plots for the insectivore GLMM. A: QQ-plots of observed quantiles versus those expected by the negative binomial distribution. Within the QQ plot are tests for deviations from the negative binomial distribution (KS test), heteroscedasticity (Dispersion test) and outliers (Outlier test). B: Standardised residuals plotted against the model predictions.

Appendix Figure S5. Activity histograms for the Collared Sparrowhawk and Brown Goshawk based on camera trap data from all sampling sites during the summer 2019 season.
