# Supplementary figures and images for: Investigating avian competition for surface water in an arid zone bioregion
Source: Ecol Evol. 2023 Aug 3;13(8):e10396. doi: 10.1002/ece3.10396 (PMC10400276; doi:10.1002/ece3.10396)

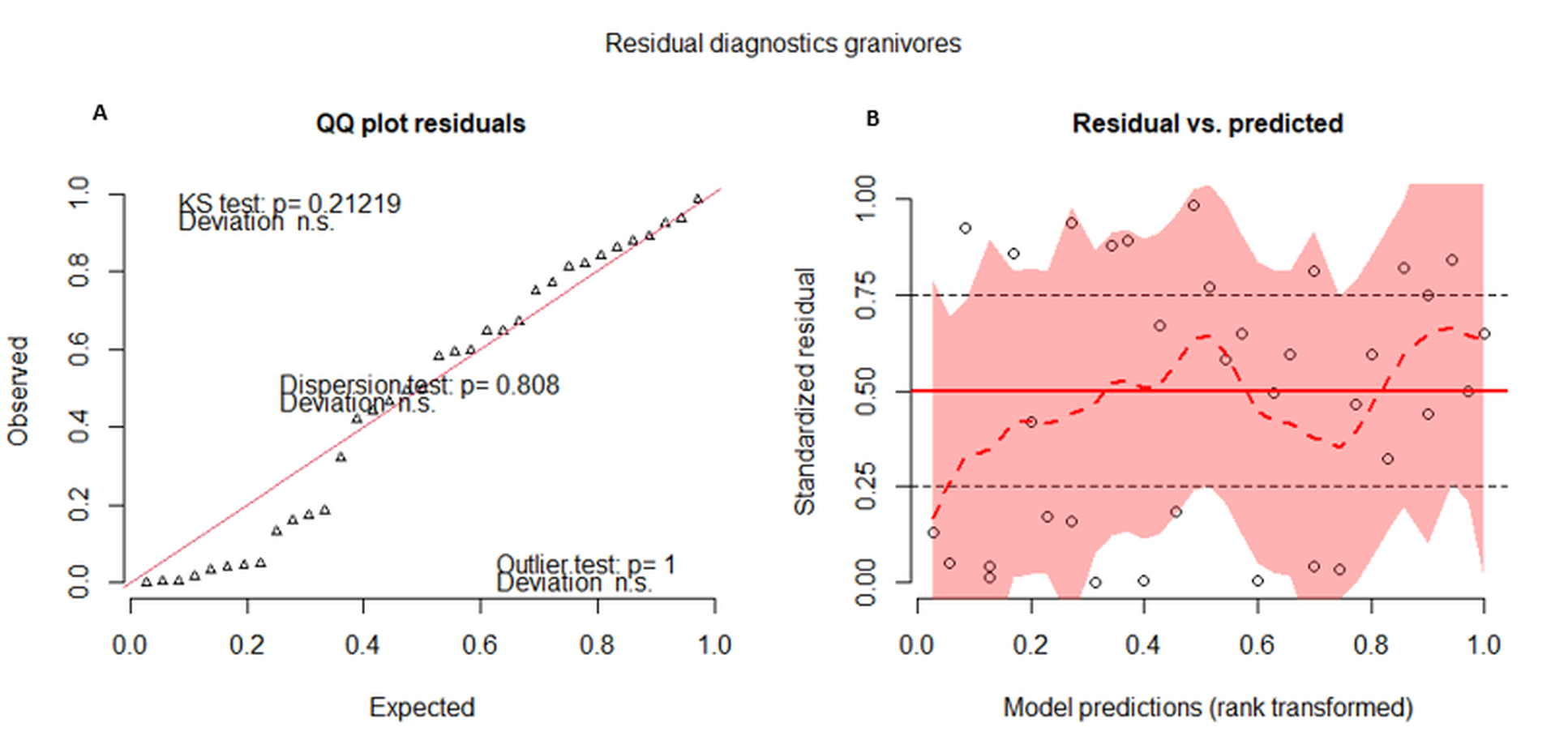

Supplement: Supplementary file 2 — Figure S1. [file ECE3-13-e10396-s001.png]

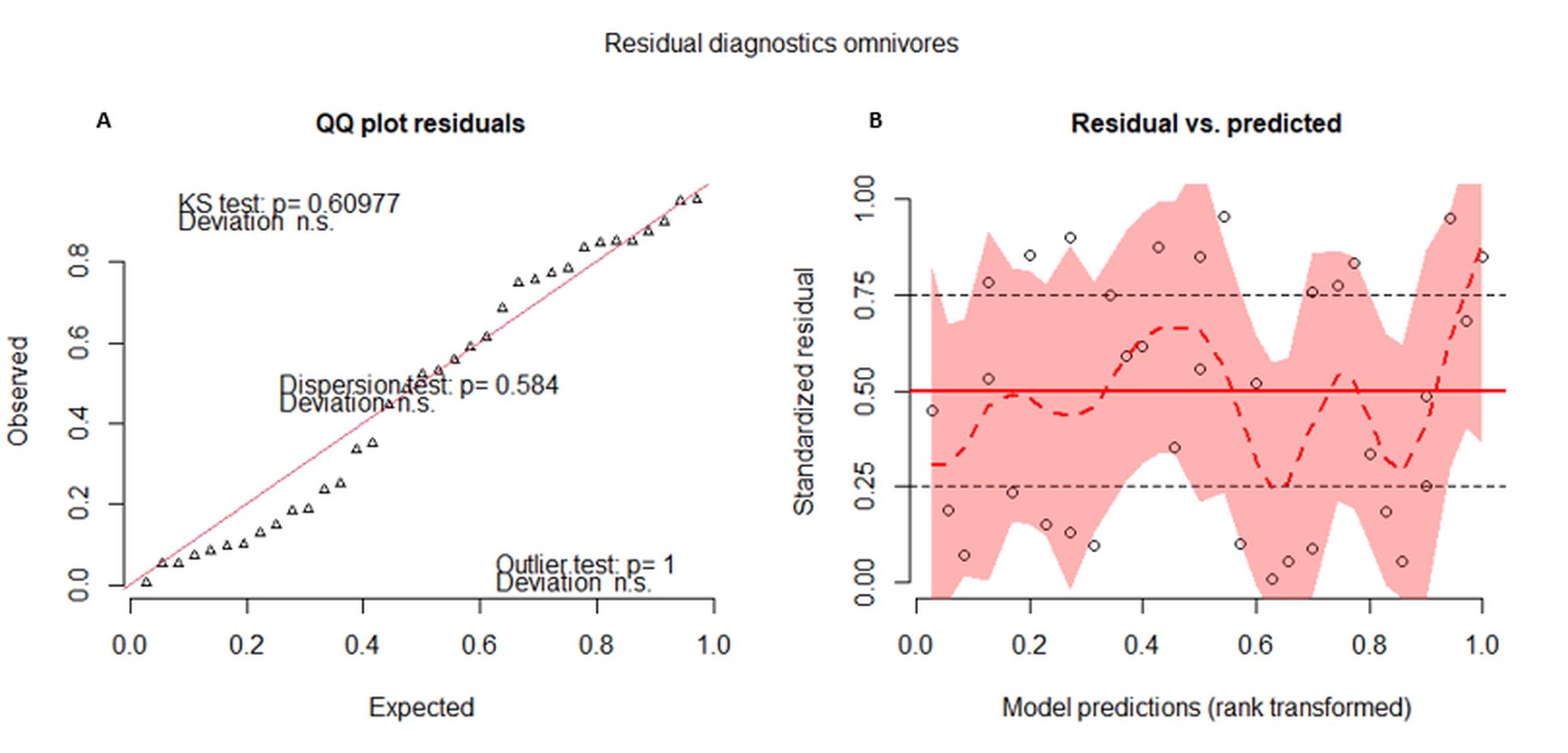

Supplement: Supplementary file 3 — Figure S2. [file ECE3-13-e10396-s002.png]

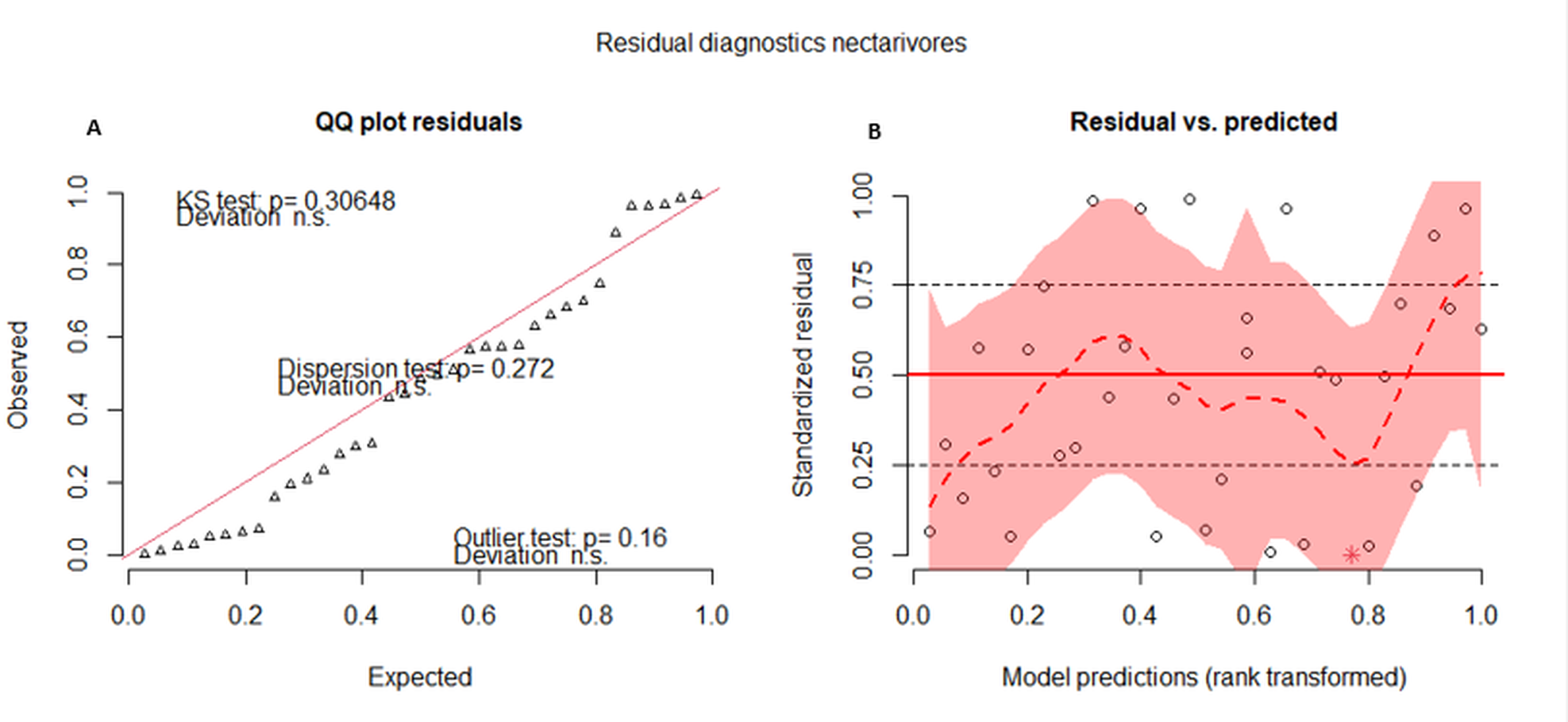

Supplement: Supplementary file 4 — Figure S3. [file ECE3-13-e10396-s003.png]

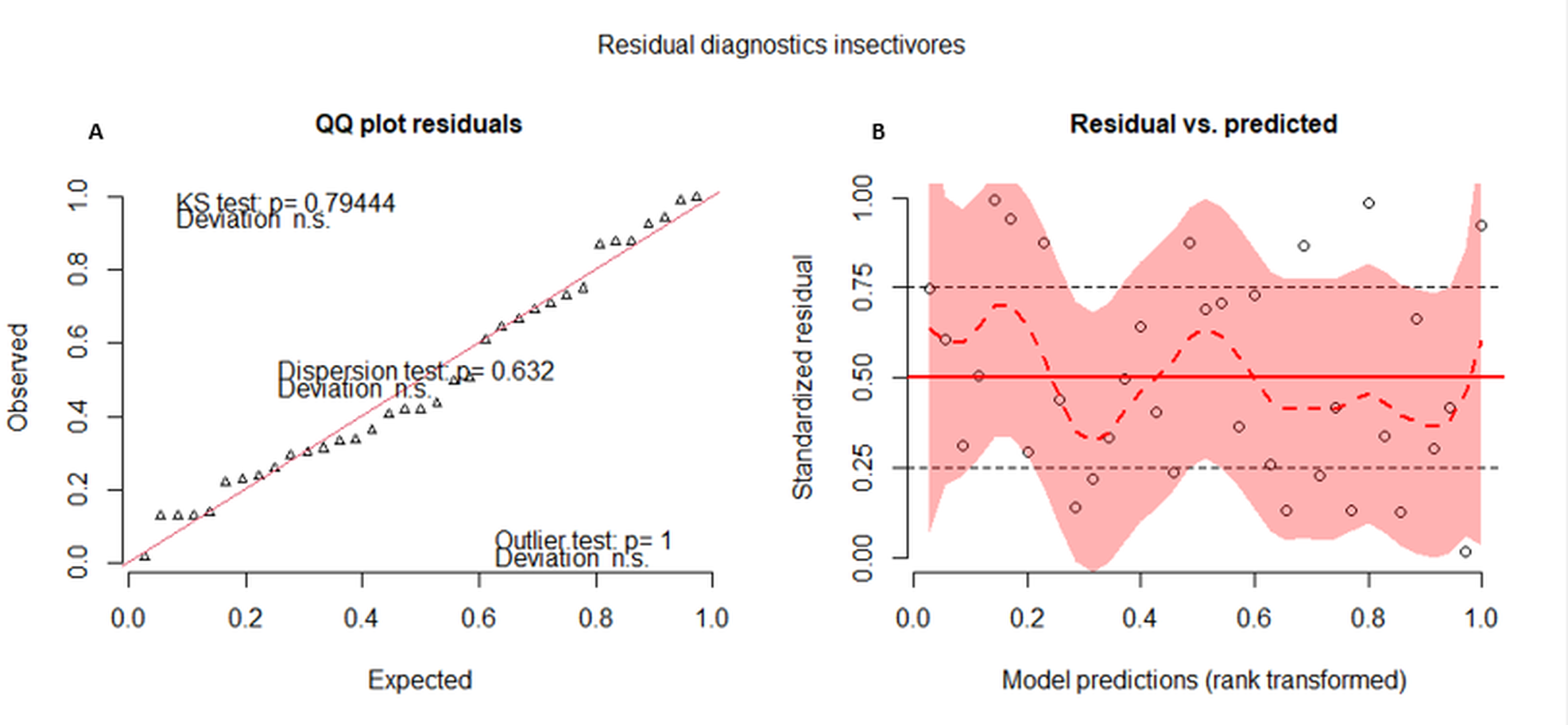

Supplement: Supplementary file 5 — Figure S4. [file ECE3-13-e10396-s004.png]

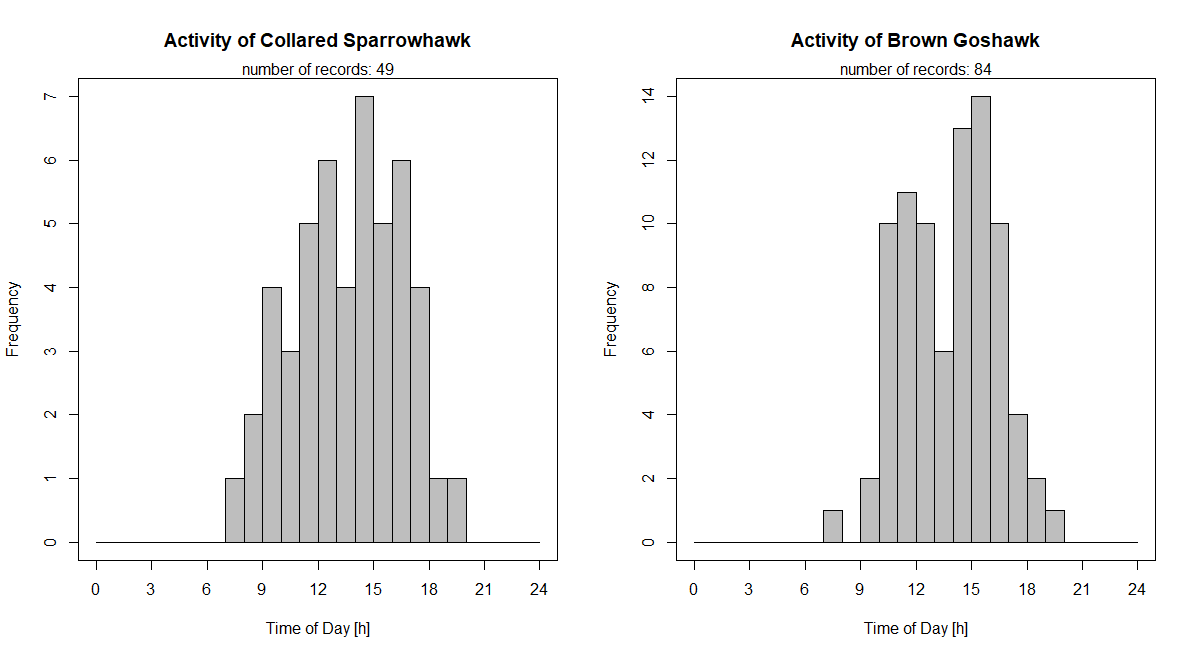

Supplement: Supplementary file 6 — Figure S5. [file ECE3-13-e10396-s006.png]
